# The study protocol for the randomized controlled trial of the effects of a theory-based intervention on resilience, social capital, psychological wellbeing, and health-promoting lifestyle in healthcare workers

**DOI:** 10.1186/s40359-023-01098-2

**Published:** 2023-03-06

**Authors:** Maryam Akbari, Mohammad Hossein Kaveh, Rosanna Cousins, Hamidreza Mokarami, Changiz Rahimi Taghanaki, Mehdi Jahangiri

**Affiliations:** 1 Department of Health Education and Promotion, School of Health, Shiraz University of Medical Sciences, Razi Ave., PO. Box 71536-75541, Shiraz, Iran; 2 Department of Psychology, Liverpool Hope University, Liverpool, UK; 3 Department of Ergonomics, School of Health, Shiraz University of Medical Sciences, Shiraz, Iran; 4 Department of Clinical Psychology, School of Educational Sciences and Psychology, Shiraz University, Shiraz, Iran; 5 Department of Occupational Health, School of Health, Shiraz University of Medical Sciences, Shiraz, Iran

**Keywords:** Randomized controlled trial, Health promotion intervention, Resilience, Social capital, Psychological wellbeing, Health-promoting lifestyle, Social cognitive theory, PRECEDE-PROCEED model

## Abstract

**Background:**

The workplace has been identified as a key determinant of health status. There is evidence of innumerable health problems among employees, particularly healthcare workers. Against this background, a holistic-systemic approach together with a good theoretical framework is required to reflect on this issue, and to support the design of effective interventions to promote the health and wellbeing of the given population. The present study aims to evaluate the effectiveness of an educational intervention in improving resilience, social capital, psychological wellbeing, and health-promoting lifestyle in healthcare workers, utilizing the Social Cognitive Theory integrated into the PRECEDE-PROCEED model.

**Methods:**

This randomized controlled trial will be performed on a large sample of the employees working in two healthcare centers in the city of Shiraz, Iran. The study will proceed with the healthcare workers of one city being given the educational intervention and the healthcare workers of the other city serving as a control group. Using a census method, all healthcare workers in the two cities will be informed of the trial and its purpose, and then invitations to join the study will be issued. The minimum sample size required has been calculated as 66 individuals in each healthcare centers. Recruitment to the trial will by systematic random sampling of eligible employees who submit an expression of interest in joining the trial, and subsequently give informed consent. Data will be collected through a self-administered survey instrument at three stages: at baseline, and both immediately and three months after the intervention. The experimental group members should participate in at least eight of the ten weekly educational sessions of the intervention and complete the surveys in the three stages. There is no educational intervention for the control group, and they simply experience some routine programs, and complete the surveys at the same three timepoints.

**Discussion:**

The findings will provide evidence for the possible effectiveness of a theory-based educational intervention to improve resilience, social capital, psychological wellbeing, and health-promoting lifestyle among healthcare workers. If the educational intervention is found to be effective, then its protocol will be exploited in other organizations to boost resilience.

*Trial registration* IRCT20220509054790N1.

## Background

The workplace has been identified as being among the main determinants of health [1]. From this perspective, a healthy workplace has the potential to bring many positive changes in employees, such as good health, higher levels of job satisfaction, and reduced absenteeism, which all contribute to improved quality of work life, and better productivity [2, 3]. Nevertheless, there is evidence of high levels of innumerable health problems among employees, particularly, healthcare workers (HCW). In particular, large sections of the working population exhibit poor mental health with evidence that excessive anxiety, emotional exhaustion, and psychological stress are rife [4]. Regarding HCW, exposure to daily crises—especially during the ongoing COVID-19 pandemic—has given rise to heavy burdens being imposed on this workforce, and high incidence rates of psychological disorders among them [5]. Any decrease in health status can reduce the quality of life (QoL) of HCW, and subsequently influence their life satisfaction [6]. These problems are harmful to the professional HCW, and also, via a reduction in their productivity in the workplace, the people they look after, and the wider society [7–9]. It follows from this that there is a business case for intervention, as well as moral and ethical reasons. There is evidence that the highest levels of sickness absence are in HCW [10]. The costs associated with sickness absence and reduced productivity are an important issue for employers, and a driver for providing resources for interventions [10–12]. Thus, a focus on HCW when designing novel, expensive interventions to improve quality of life in the working population is an effective approach.

A wide variety of factors can shape employees’ mental health. At the individual level, studies have established that many HCW might lack resilience and stress management skills [13]. At the interpersonal level, no support from coworkers or family members might contribute to the emergence and exacerbation of the problems [14]. The quality of communications between managers and employees, along with perceived justice, have also been reported to shape mental health in employees at the organizational level [15]. It follows from this that multiple interventions are often required for multi-causal health problems [16]. Therefore, the effectiveness of mental health-promoting interventions in the workplace depends on adopting a holistic-systematic and evidence-based approach together with a good theoretical framework to promote the health and wellbeing of the given population. Characterized by having an ecological-holistic approach, the PRECEDE-PROCEED model provides a suitable roadmap for this purpose [17]. This PRECEDE-PROCEED model represents a process to change behavior, and a structure to systematically apply theories and concepts in order to plan and evaluate the success rate of educational health promotion programs regarding behavior change [18–22]. The PRECEDE-PROCEED model has been successfully used in several workplace-based health promotion programs [17] which also strongly supports its suitability for the problem being addressed in the proposed research. As a systematic planning framework, it comprises eight phases for analyzing a problem, designing an intervention, and finally evaluating a program [21]. In line with all health-promotion interventions, promoting the QoL is the ultimate goal of the PRECEDE-PROCEED model [22]. The eight phases of the model are listed in column 1 of Table 1.

In the present study, the groundwork for the evidence-based targeted intervention measures that will be implemented and evaluated was provided by a review of the literature to assess the social and epidemiological background of similar HCW populations. This approach implemented the first three phases of the PRECEDE-PROCEED model and was essential to achieve a logical framework for planning the health-promoting intervention and identifying its components. The social, epidemiological, behavioral, and environmental assessment provided by the literature review found evidence that the mental health and QoL of HCW was negatively by their work, particularly since the outbreak of the COVID-19 pandemic [23, 24]. It also confirmed that QoL can be affected by one’s health conditions [25–27]. Psychological wellbeing (PWB) is accordingly one of the determinants of the QoL among employees [28], and it is strongly influenced by one’s psychosocial environment and behavior. Here, wellbeing refers to a concept that defines the quality of work life, as a main determinant of productivity in individuals, organizations, and society [29]. People with low wellbeing may thus evaluate events and situations in their lives as adverse ones, and undergo negative emotions, e.g., anxiety, depression, and anger [30].

With respect to environmental determinants of health, evidence suggests that the conditions related to the psychosocial environment affect employee health more than the physical setting characteristics [31]. Social capital is one of the major components of the psychosocial environment at work [32]. The concept of social capital in the workplace represents the attitudes and values ​​among members, mutual action, respect, and trust between coworkers, collective action, contribution to networks, and trust in honesty among managers at work [33]. The workplace is correspondingly an essential source of social capital for many people, which provides mutual support and gives meaning to life [34]. Evidence indicates that social capital is significantly correlated with health outcomes, organizational outcomes, and a health-promoting lifestyle (HPL) in employees [35]. In the dimension of organizational outcomes, social capital can have a greater impact on job success, knowledge sharing, increased communication, and improved chances of organizational survival [36, 37]. Social capital also seems to be associated within the HPL dimension. If being deprived of healthy lifestyle can result in depression, absenteeism, and low productivity at work, then keeping employees healthy and productive is crucial for boosting productivity at the organizational level [38]. Likewise, there are reciprocal relationships between social capital, PWB, and resilience. Resilience denotes a set of protective strategies used by individuals in situations they once encountered with difficulty or deprivation. Such strategies change the way a person reacts to difficulties, and they often integrate both internal and external factors in a positive direction, with favorable outcomes, such as maintaining or returning to health. Demonstrating resilience also leads to positive mental health or prevention of some negative consequences [39]. Recent viewpoints consider resilience as a capacity that can be consciously developed through learning processes [40–42]. Similarly, it has been argued that resilience training can improve mental health and wellbeing outcomes for workers [43]. Resilience accordingly plays a leading role in helping employees to successfully reach adaptation, handle emotional pressure, develop effective coping strategies, and improve their wellbeing [39]. Accordingly, the main goals of developing social capital, PWB, and resilience in the workplace bear a resemblance. In other words, one of the hypotheses arising from such facts is that the elevation in resilience skills improves social capital, and both promote PWB. In such a context, a HPL will be more encouraged by bolstering the psychological-social atmosphere in the workplace [30, 44].

The third phase of the PRECEDE-PROCEED model is an educational and ecological assessment, wherein the determinants of behavior and environment are typically examined in three categories: predisposing, reinforcing, and enabling factors. Here, predisposing factors include knowledge, attitudes, self-efficacy, outcome expectations, and outcome values; reinforcing factors are perceived social or organizational support, and enabling factors consist of having access to and support of resources and personal skills [22–24]. In different phases of applying the PRECEDE-PROCEED model, especially in the third phase, the specific theories of behavior change related to the research problem and setting can be used [20, 45], which help in augmenting the internal validity of the study. In light of this, the Social Cognitive Theory (SCT) is suitable for the analysis of the problem in the PRECEDE-PROCEED model. This theory can explain human behavior in terms of having three-way causality (individual, environmental, and behavioral) [46, 47], whose interactions can produce behavior change [48, 49].

The fourth phase of the PRECEDE-PROCEED model includes a review of educational programs, policies, and procedures within an organization, and ultimately the intervention program is developed. During the fifth phase, the proposed intervention program is implemented among employees, then in phases six to eight comprise process, impact, and outcome evaluations [21, 22, 24, 50].

To date, interventions to improve resilience towards better health outcomes have lacked a longitudinal element, good outcome measures, and comprise other design issues [51]. Similarly, despite strong assertions that improving social capital can improve health, there is a dearth of intervention studies to examine these claims [52]. In general, it seems that the PRECEDE-PROCEED model integrated into the three SCT factors (individual, environmental, and behavioral) can be exploited as a suitable theoretical framework for the design and evaluation of mental health-promoting interventions among HCW in a longitudinal RCT. Therefore, the aim of this study is to determine the effects of an evidence-based educational program on resilience, social capital, PWB, and HPL in HCW, using the SCT integrated into the PRECEDE-PROCEED model.

### Research hypotheses

1. The change mean scores of the study constructs (viz. resilience, social capital, PWB, HPL, and resilience) among HCW in the experimental and the control groups are different after the intervention is completed.
2. There is a significant correlation between resilience and social capital, resilience and PWB, and resilience and HPL in both experimental and control groups.
3. There is a significant correlation between the constructs of the SCT and the variables of resilience, social capital, PWB, and HPL.

## Methods

### Study design, setting and participants

This quasi-experimental study is a non-blinded randomized controlled trial (RCT) (Code: IRCT20220509054790N1) with assessments at baseline, immediately after the intervention, and three months after the intervention. This study was approved by the Ethics Committee in Medical Research affiliated to Shiraz University of Medical Sciences, Shiraz, Iran.

Recruitment will be from two large healthcare centers in the northern and southern parts of the city of Shiraz, in southern Iran (namely, Wal-fajr and Enghelab). Permission to undertake the study in these two healthcare centers was sought as they are comparable in their cultural and socio-economic characteristics. This method has previously been used as a means of preventing information exchange between the participants in the experimental and control groups [53]. The two healthcare centers will be randomly assigned to intervention and control settings following recruitment. Using the MedCalc software and the following formula:

$$n \ge \frac{{(Z_{{1 - {\raise0.5ex\hbox{$\scriptstyle a$} \kern-0.1em/\kern-0.15em \lower0.25ex\hbox{$\scriptstyle 2$}}}} + Z_{{1 - \beta }} )\left( {\sigma _{1}^{2} + \sigma _{2}^{2} /r} \right)}}{{(\mu _{1} - \mu _{2} )^{2} }}$$

The sample size is estimated to be 66 participants in each group. Considering the 5% loss of observations, the final sample size will be determined to be 69 in each group.

The study has been advertised in the two healthcare centers, and information about the study distributed to all HCW. Inclusion criteria are being employed at the healthcare center, aged 25–60 years, and no self-reported history of severe physical or mental illness. (This will also be checked again at the community survey stage). Exclusion criteria are severe physical or mental illness, not giving informed consent, reluctance to continue the study/not participating in either intervention and/or surveys. For sampling, first, an alphabetical list of the names of all HCW in both centers is prepared. Thenceforth, the samples will be selected in a randomized manner. If a person is not eligible, or does not give written informed consent, then they will be replaced with the next one available on the list will be replaced. Baseline data will then be collected from all participants. There are no incentives for participating in the research, but also no costs because data collection will be during working time. All participants who complete the study will be given a certificate of participation in the study.

**Fig. 1:**
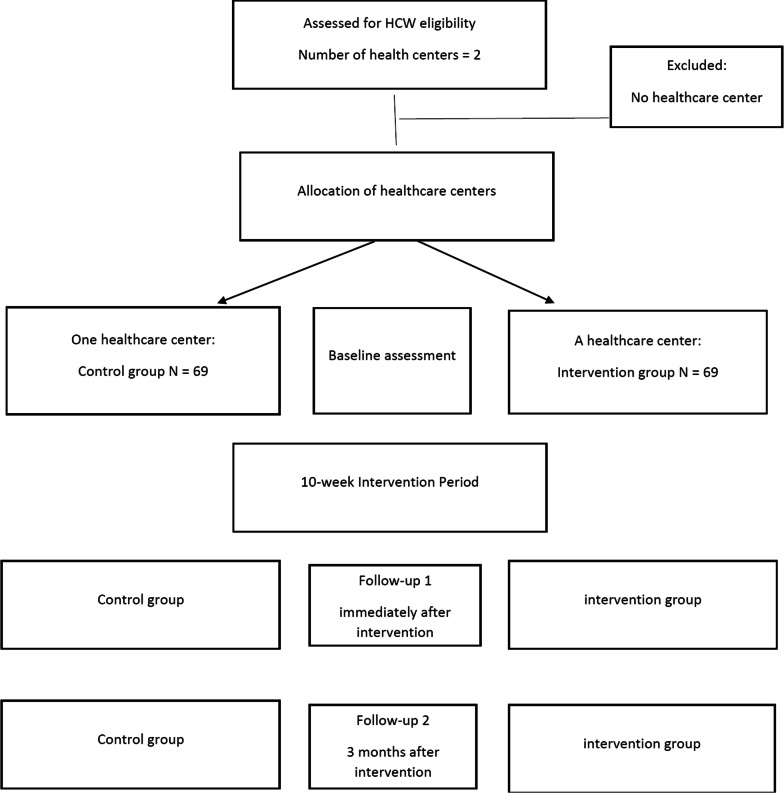
CONSORT flow diagram for enrolment and randomization in the study

**Fig. 2:**
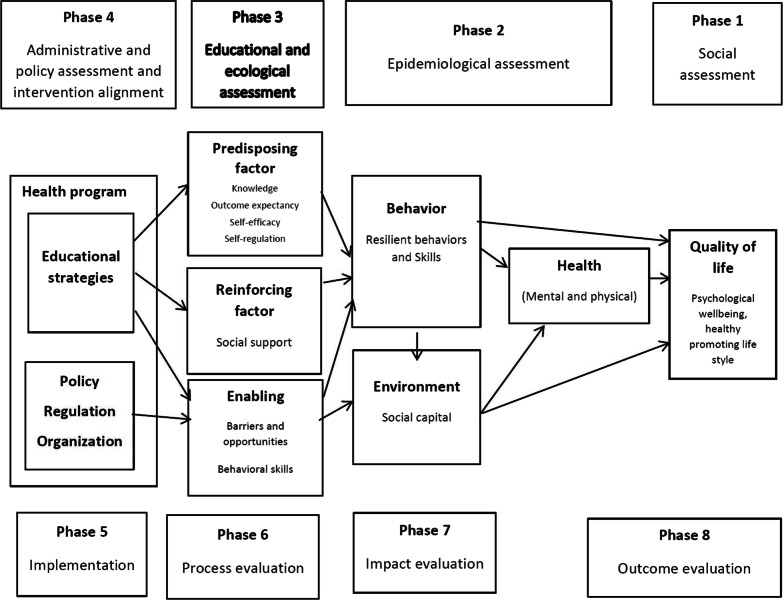
Application of the PRECEDE-PROCEED model

A flow diagram of the RCT protocol is presented in Fig. 1. The control group will have no intervention. During the 10-week intervention period participants in the control group healthcare center will be offered routine staff training opportunities. Participants in the intervention group will receive the intervention described below. The logic model used for the intervention planning and evaluation—the PRECEDE-PROCEED model—is illustrated in Fig. 2. Table 1 shows the key research questions and the data sources according to the model phases.

**Table 1:** Phases 1–8 of PRECEDE-PROCEED model: the main questions to be addressed and the sources of data

Phase	Main questions to be addressed	Data sources
1. Social assessment	What are the demographic characteristics of the study participants? What is the QoL status of HCW?	Literature review
2. Epidemiological assessment	What are the behavioral risk factors affecting psychological well-being? What are the environmental causes of the health concerns? How is the psychological well-being of the participants?	Literature review Community survey (Measuring psychological wellbeing, health-promoting lifestyle, resilience, and social capital)
3. Educational and ecological assessment	What are the predisposing factors? (Knowledge, self-efficacy, outcome expectation, outcome expectancy, and self-regulation) What are the reinforcing factors? (Social support) What are the enabling factors? (Behavioral skills)	Literature review Community survey (Developing a tool for measuring determinant of resilience based on SCT)
4. Administrative and policy assessment and intervention alignment	What are the procedures, resources, and other capacities in the organization? Which policies, rules, and organizational aspects restrain changes in enabling factors? What are the factors affecting the implementation of the intervention? (Understanding barriers and opportunities)	Interview with key informant of organization
5. Implementation	What are the health promotion strategies per educational objective? Are strategies in line with the aims? What is the deadline for the implementation of strategies? Which resources are required?	Educational intervention
6. Process evaluation	What are the process indicators?	Group discussion Polls form
7. Impact evaluation	What are the impact indicators?	Community survey
8. Outcome evaluation	What are the outcome indicators?	Community survey

### Intervention program

The evaluation program will be based upon the the evaluations from the first three phases and a consideration of the characteristics of the target population. Theories of behavior change such as the SCT we are using to support our educational program, show the focus of the intervention program and propose useful strategies to achieve the goals of change [54]. For example, the concept of self-esteem, and ways to increase it, will be taught to improve self-efficacy. Similarly, the intervention group will be trained with thinking traps and recognizing their thoughts to improve self-regulation skills, in order to control their emotions, as well as speed skills to monitor their emotions. Although there is some diversity in terms of education duration and topics [55] the available evidence suggests that empowering employees in the field of resilience skills is one of the important components of health promotion programs [56]. The effectiveness of an educational program is also dependent upon the application of educational theories, which can support the ability of the material to change the behavior of the target population [57]. Theories that explain adult learning suggest that collaboration and active involvement in the educational sessions are required, as well as positive reinforcement by timely feedback [58]. In view of this, some techniques, such as small-group discussions, role-plays, and questions and answers will be used to support good face-to-face teaching-learning practices. The educational sessions will also include video clips, podcasts, pamphlets and other purposeful handouts to support assimilation of the materials both during the sessions and as homework. This can help increase the learning of more contents during the education process [59, 60].

Drawing on intervention studies that have published their experiences, it seems that an educational program consisting of 8–10 sessions, should be effective in developing basic resilience skills. Similarly, other interventions with educational training in weekly sessions of 60–90 min have been found to have sustainable positive outcomes [53]. Thus, the contents of the intervention program will proceed with 10 sessions, each approximately 60–90 min, with the contents shown in Table 2.

**Table 2:** Contents of the educational intervention program sessions

Sessions	Contents
1	Introduction. familiarization with the research objectives, groups, and participants
2	Resilience, familiarity with emotions
3	Cognitive traps
4	Cognitive traps
5	Problem-solving skills
6	Speed skills to control emotions
7	Effective communication skills
8	Effective communication skills
9	Self-esteem and self-efficacy skills
10	Self-esteem and self-efficacy skills

### Data collection and instruments

Data will be collected at three time points from both the intervention group and the control group. A pen and paper survey will be distributed at dedicated times during the working day, according to HCW shift. Time will be set aside for participants to complete their survey, and post anonymously in a sealed collection box. made up for the following instruments:

### Demographic information

A researcher-made questionnaire will be used to collect information regarding participants sex, age, education level, marital status, and history of illness, seeking psychiatric support and taking medication. (Participants who report chronic illness needing medication, or consultations with a psychiatrist who give a positive answer at any of the three time points will be excluded from the study.)

*Social Capital at Work* [61]. This measure is made up of eight items that together assess the cognitive and structural components of social capital at work. Items explore mutual respect and trust between colleagues and a supervisor, reciprocity, participation in networks and collective action. An example is: “People feel understood and accepted by each other”. Answers to the statements are given using a five-point agreement scale where 1 = fully disagree, to 5 = fully agree, and low scores will indicate low social capital. This questionnaire has been reliably used in Iran [62].

*Resilience at Work Scale* [42]. The scale authors assert that resilience is a capability and that the scale supports intervention to support employees develop strengths in these areas to enable them to cope with work challenges. It comprises 20 items that assess seven aspects of resilience at work: living authentically (3 items), finding one’s calling (4 items), maintaining perspective (3 items), mastering stress (4 items), interacting cooperatively (2 items), staying healthy (2 items) and building networks (2 items). Each item is rated using seven-point agreement scale ranging from 1 = completely disagree to 7 = completely agree, and high scores indicate higher resilience. Previous studies have confirmed its reliability [39]. This will also be confirmed in this study through Cronbach’s alpha coefficient and test-retest method.

*Resilience Skills Questionnaire*. This is a 17-item researcher-made questionnaire based on three key constructs of SCT: self-efficacy, self-regulation and reinforcement from social support. Participants respond to each item using a 5-point agreement scale where 1 = strongly disagree, to 5 = strongly agree, and higher scores will indicate better status in each construct. A pilot study in a sample of the statistical population indicated the RSQ has good psychometric properties. This study will confirm its reliability through Cronbach’s alpha coefficient and test-retest method.

*Psychological Wellbeing Scale* [63]. There are various versions of scales to measure the six dimensions of PWB originally identified by Ryff [64]: environmental mastery, personal growth, purpose in life, self-acceptance, autonomy, and positive relations. In this study we used an 18-item version originally designed by Ryff and Keyes [65] and validated in an Iranian sample by Khanjani et al. [63]. There are three items for each of the six dimensions; participants will rate themselves on a six-point agreement scale where 1 = strongly disagree to 6 = strongly agree. Scores on items that are negatively phrased will be reversed so that the higher the total score, the greater the participant’s well-being. Dimensional scores can also be calculated (range 3–18).

*Health-Promoting Lifestyle Profile* [66]. This 52-item questionnaire assesses the extent to which adults engage in health behaviors associated with six dimensions: health responsibility, spiritual growth, physical activity, nutrition, stress management and interpersonal relations. Participants will use the original a four-point frequency scale where 1 = never, 2 = sometimes, 3 = often, to 4 = routinely. The reliability and validity of a Persian version of the scale have been measured by Mohammadi Zeidi et al. [67].

### Data analysis

Descriptive and inferential statistical analyses will be conducted using IBM SPSS-Amos software. The Kolmogorov-Smirnov test will be correspondingly applied to check the normality of the data. If the data are normally distributed then t-tests, paired-samples t-tests, and analysis of variance (ANOVA) will be operated as needed. Analysis of covariance (ANCOVA) will be employed to investigate the difference in the mean scores of the experimental and control groups in terms of the study variables at the pre- and post-intervention stages. If the data are not normally distributed, non-parametric analysis will be used. In this case, the Mann-Whitney U test will be used to compare two groups before and after the intervention, and the Wilcoxon signed-rank test will be applied to compare each group at the pre- and post-intervention stages, and the Kruskal–Wallis test will be exploited for the between- and within-group comparisons.

In addition, structural equation modeling will be utilized to analyze and evaluate the fit of the model.

## Discussion

The present study will be the first attempt to examine the effectiveness of an educational intervention, developed based on the SCT and integrated into the PRECEDE-PROCEED model, in improving resilience, social capital, PWB, and HPL among HCW. The ultimate goal of the study is present a workplace intervention that will boost resilience, social capital, PWB, and HPL for the benefit of employees and employers. This evidence-based study has several strengths, as we discuss below.

The extant literature includes several intervention studies to improve workplace health. Nevertheless, there are contradictory results. Whilst some interventions have confirmed the effectiveness of resilience training [43], other studies have considered it to be insignificant. The lack of consensus may be due to the quality of the intervention program, or the study settings or the statistical population involved in the study, or the measures used, or a combination of all of these. Workplace interventions to improve employee health and quality of life are complex. There is a need to use established theories and principles, as well as a health promotion/illness-prevention framework to support the essential risk assessment process [68], yet the literature suggests this is not so. This study is a theory-based intervention based on an established health promotion framework, using delivered using theory-based educational practices. This is a strength of this study.

This study will be an RCT—the most powerful tool in experimental research to investigate the effectiveness of an intervention [69, 70]. Conducting a randomized interventional study accordingly makes it possible to examine the effects of the intervention on the outcomes among individuals in experimental and control groups [71].

Another strength of this study is that the contents of the educational sessions will be delivered to the participants face-to-face [53] and using a variety of activities and media to support assimilation of the knowledge materials both during the sessions and as homework. This can help increase the learning of more contents during the education process [59, 60].

The educational sessions have as their focuses the main concepts found to influence employee health in the modern workplace. The ten sessions include educational practices to boost resilience skills, and to adopt healthy behaviors. The primary aim is to support HCW who need to be mentally resilient and flexible so that they can provide good services to others in society [72]. Resilience is one of the effective factors in employee performance [73]. Ultimately, the study results will have implications in practice for promoting resilient behaviors in HCW. Then, assuming this intervention is found to be effective in this group of employees, it can be put to the test in other organizations and on their employees. Certainly, there is a business case for intervention, and interventions in the workplace support public health, and mental health agendas [68]. Thus, a focus on HCW when designing novel, expensive interventions to improve quality of life in the working population is an effective approach.

A potential limitation of this study is that it was not possible to run a small pilot of the educational program in advance of the full RCT. This, however, is typical of comprehensive intervention studies that use RCT with a longitudinal element to assess sustainability of the learning. Members of the research team have experience of educational interventions in other context, and our preparatory work indicated that the intervention is feasible. Similarly, it is unlikely that we will be able to separate out the different needs for the difference types of HCW in this study. A challenge with pilot studies for complex projects, and for accounting for all job roles are threefold: resource costs, time, and sufficiency of participants. We are confident, however, that we will have enough of all three to meet the aim and objectives of the project.

## Conclusion

The present study describes a protocol for an RCT to evaluate the effectiveness of an intervention based on the SCT using the PRECEDE-PROCEED health promotion model to boost resilience, social capital, PWB, and HPL among the HCW in the city of Shiraz, Iran. The methodology is robust. The results will provide information about the effectiveness of theory-based interventions on promoting resilient behaviors and the potential of these interventions to influence outcomes, such as social capital, PWB, and HPL in future research.

## Data Availability

Not applicable. The manuscript does not report data. The datasets subsequently generated and/or analyzed during the current study may be made publicly available following conclusion of ongoing research. Requests for data may be made at any time to the corresponding author.

## Abbreviations

HCW: Healthcare workers
SCT: Social cognitive theory
PWB: Psychological wellbeing
QoL: Quality of life
HPL: Health-promoting lifestyle
CONSORT: Consolidated standards of reporting trials
RCT: Randomized controlled trial
